# Asia-Pacific Perspectives on the Role of Continuous Glucose Monitoring in Optimizing Diabetes Management

**DOI:** 10.1177/19322968231176533

**Published:** 2023-05-26

**Authors:** Stephen Twigg, Soo Lim, Seung-Hyun Yoo, Liming Chen, Yuqian Bao, Alice Kong, Ester Yeoh, Siew Pheng Chan, Jeremyjones Robles, Viswanathan Mohan, Neale Cohen, Margaret McGill, Linong Ji

**Affiliations:** 1 Charles Perkins Centre, Faculty of Medicine and Health, University of Sydney, Sydney, NSW, Australia; 2 Department of Endocrinology, Royal Prince Alfred Hospital, Sydney, NSW, Australia; 3 Department of Internal Medicine, Seoul National University Bundang Hospital, College of Medicine, Seoul National University, Seongnam, South Korea; 4 Department of Internal Medicine, Korea University Anam Hospital, Seoul, South Korea; 5 Tianjin Key Laboratory of Metabolic Diseases, Chu Hsien-I Memorial Hospital and Tianjin Institute of Endocrinology, Tianjin Medical University, Tianjin, China; 6 Department of Endocrinology and Metabolism, Shanghai Jiao Tong University School of Medicine, Affiliated Sixth People’s Hospital, Shanghai, China; 7 Department of Medicine and Therapeutics, The Chinese University of Hong Kong, Shatin, Hong Kong; 8 Diabetes Centre, Admiralty Medical Centre and Division of Endocrinology, Department of Medicine, Khoo Teck Puat Hospital, Singapore; 9 Department of Medicine, University of Malaya Medical Centre, Kuala Lumpur, Malaysia; 10 Section of Endocrinology, Diabetes and Metabolism, Department of Internal Medicine, Chong Hua Hospital, Cebu, Philippines; 11 Dr. Mohan’s Diabetes Specialities Centre and Madras Diabetes Research Foundation, Chennai, India; 12 Baker Heart and Diabetes Institute, Melbourne, VIC, Australia; 13 Central Clinical School Faculty of Medicine and Health, Diabetes Centre, Royal Prince Alfred Hospital, The University of Sydney, Sydney, NSW, Australia; 14 Peking University Diabetes Center, Department of Endocrinology and Metabolism, Peking University People’s Hospital, Beijing, China

**Keywords:** Asia, clinical practice patterns, continuous glucose monitoring, diabetes mellitus, Oceania, time-in-range

## Abstract

Diabetes is prevalent, and it imposes a substantial public health burden globally and in the Asia-Pacific (APAC) region. The cornerstone for optimizing diabetes management and treatment outcomes is glucose monitoring, the techniques of which have evolved from self-monitoring of blood glucose (SMBG) to glycated hemoglobin (HbA1c), and to continuous glucose monitoring (CGM). Contextual differences with Western populations and limited regionally generated clinical evidence warrant regional standards of diabetes care, including glucose monitoring in APAC. Hence, the APAC Diabetes Care Advisory Board convened to gather insights into clinician-reported CGM utilization for optimized glucose monitoring and diabetes management in the region. We discuss the findings from a pre-meeting survey and an expert panel meeting regarding glucose monitoring patterns and influencing factors, patient profiles for CGM initiation and continuation, CGM benefits, and CGM optimization challenges and potential solutions in APAC. While CGM is becoming the new standard of care and a useful adjunct to HbA1c and SMBG globally, glucose monitoring type, timing, and frequency should be individualized according to local and patient-specific contexts. The results of this APAC survey guide methods for the formulation of future APAC-specific consensus guidelines for the application of CGM in people living with diabetes.

## The Journey of Glucose Monitoring in People Living With Diabetes

An estimated 537 million individuals globally, or 10.5% of the world population, have been affected by diabetes.^1^ In 2021 alone, around 6.7 million people worldwide died from diabetes.^1^ In 2017, the disease was also the fourth leading cause of disability across the globe.^2^

Glycemic control has been a well-accepted cornerstone of diabetes management since the Diabetes Control and Complications Trial in the 1980s-1990s,^3^ which clearly reinforced the clinical utility of glycated hemoglobin (HbA1c) as the gold standard for glucose monitoring and for predicting the risk of complications (especially microvascular) associated with diabetes.^4^ HbA1c, which reflects glycemic control over 2 to 3 months, is the only glycemic parameter that has been evaluated prospectively and found to be strongly predictive of chronic diabetes complications.^5,6^ Although intra-individual HbA1c correlates with mean glucose over time, HbA1c is an indirect measure that may underestimate or overestimate the average glucose level because it cannot reflect glycemic variability or hypoglycemia.^5^ Furthermore, HbA1c is influenced by conditions involving the turnover of red blood cells and is unreliable in patients with comorbidities such as anemia, hemoglobinopathies, chronic renal insufficiency, and severe decompensated liver diseases.^6,7^ Race and ethnicity may also have an influence on HbA1c, with blacks, Hispanics and Asians noted to have elevated HbA1c levels compared with their white counterparts with type 2 diabetes mellitus (T2DM).^8^ Therefore, to optimize glycemic monitoring, the American Diabetes Association (ADA) and other international organizations have proposed other measures such as self-monitoring of blood glucose (SMBG) and continuous glucose monitoring (CGM).^5,6,9-11^

After the advent of blood glucose strips for SMBG in the 1960s, a series of technological improvements over the late 1970s-2000s allowed the initiation and increasingly easier use of SMBG in home settings.^12-14^ Despite these advances, the accuracy of SMBG may be dependent on the pricking and blood application techniques and the type of glucose meter, which in turn may be influenced by multiple factors such as oxygen saturation, temperature, interfering substances, counterfeit test strips, and improper storage of strips.^15,16^ Furthermore, the patient/carer inconvenience of drawing blood for sampling and the pain associated with finger pricking are significant impediments to SMBG. There is also a wide variation in the accuracy of glucose meters available in the market. According to a study that compared 17 point-of-care glucose meters, the mean absolute relative difference of the assessed meters ranged from 5.6% to 20.8%.^17^ This highlights the need for blood glucose meters to adhere to the accuracy assessment, design verification, and performance validation guidelines outlined by ISO 15197:2013.^18^

The approach to glucose monitoring was later revolutionized by the advent of CGM, whereby interstitial fluid glucose was measured with a subcutaneous sensor, transmitter, and receiver (monitor). In 1999, the first CGM system was approved for use in diabetes. A physical cable connected the sensor and receiver, and stored data became available to the health care provider (HCP) after a three-day wear period. Subsequently, device updates allowed real-time monitoring and data viewing by users, with alerts for hypoglycemia and hyperglycemia.^12,14,19^ One key limitation of this system was the need for daily capillary glucose calibrations. In the succeeding years, new CGM systems with longer wear periods (up to 180 days) became available. Some systems permitted data transmission to mobile devices, while others were integrated into automated insulin pumps and smart insulin pens. Flash glucose monitoring was introduced, wherein users could scan the receiver over the sensor to view glucose levels and trends. This CGM system eliminated the need for capillary glucose calibrations. Over time, sensor technology improved in terms of lifetime, less warm-up time, detection methods, smaller size, reduced foreign body reactions, and greater specificity and accuracy, and connectivity, as it incorporated Bluetooth-based, real-time transmission and real-time low and high glucose alarms and glucose excursion trends.^12,14,19^ Remote data transmission via Bluetooth enables spouses and caregivers of elderly people with diabetes and parents of children with diabetes to monitor and receive automated alerts.^20^

Presently, the available CGM devices can be categorized according to their intended use:

- Professional—applied in the clinic and provide blinded or unblinded data for use by the HCP
- Personal—display unblinded data, either continuously, as in real-time CGM (rtCGM), or when swiped by a reader or app on a phone, as in flash or intermittently scanned CGM (isCGM).^15,19,21,22^

Recent real-world data from high-income Western populations demonstrate that about one-third of people with type 1 diabetes mellitus (T1DM) use CGM. As CGM uptake has been increasing steadily across the world,^23^ the International Consensus on Time in Range (TIR) developed and standardized 14 CGM metrics in 2019, with the goal of global alignment.^6^ However, disparities in CGM adoption and use have been shown to occur according to race/ethnicity and socioeconomic status.^23^ Regional differences may also exist in terms of achievement of CGM target metrics.^24^ Hence, these findings warrant a closer evaluation of regional and country-specific patterns of CGM use.

## Regional Perspectives on Glucose Monitoring

### Burden of Diabetes in the APAC Region

Diabetes is highly prevalent in the Western Pacific Region (WPR) and Southeast Asia (SEA).^1^ Table 1 shows the burden of diabetes in Asia-Pacific (APAC) countries based on the 2021 data from the International Diabetes Federation.^1^ China and India have the highest diabetes populations in the world and collectively account for 40% of all people with diabetes.^1^ In 2017, the absolute number of cases of T1DM were estimated to be the highest in Asia,^25^ while an epidemic of T2DM was also arising in the region.^26^ The diabetes burden in the APAC region is proposed to be driven by intergenerational, intrauterine, and epigenetic modifications from industrialization, urbanization, and the corresponding lifestyle changes.^26^ These risk factors have displayed an increased propensity to cause diabetes in South Asia and the WPR, compared with Western populations.^26^ Due to interregional and intraregional differences in health care models, policies, and resources, as well as ethnic and cultural differences in glycemic patterns in people with diabetes, international guidelines may not be completely applicable to APAC. Hence, the existing global standards of care in diabetes, including those for glucose monitoring, need to be tailored to the APAC setting.

**Table 1.** The Burden of Diabetes in APAC Countries as Per 2021 IDF Data.^1^

Country	Individuals with diabetes, in 1000s (20-79 years)	Age-adjusted prevalence of diabetes (%)
Australia	1491.80	6.4
China	140 869.60	10.6
Hong Kong	686.00	7.8
India	74 194.70	9.6
Japan	11 005.00	6.6
Malaysia	4431.50	19.0
Philippines	4303.90	7.1
Singapore	711.80	11.6
South Korea	3511.80	6.8

APAC, Asia-Pacific; IDF, International Diabetes Federation.

### APAC Expert Panel Meeting for Optimizing Regional Glucose Monitoring Practices

On October 30, 2021, the APAC Diabetes Care Advisory Board, formed by 15 diabetes experts from nine countries in the APAC region (Australia, China, Hong Kong, India, Japan, Malaysia, the Philippines, Singapore, and South Korea), convened for the first time to gather insights and guidance on the utilization of CGM systems and optimization of glucose monitoring and diabetes care in APAC. Prior to the meeting, a survey was conducted among the 15 experts to understand the real-world insights on glucose monitoring patterns and practices. The compiled results from the survey and the available literature formed the background of discussions during the meeting.

We present here the findings from the survey and the advisory board meeting, along with supporting literature for optimizing glucose monitoring in APAC.

### Glucose Monitoring Patterns in APAC

There is a lack of literature on glucose monitoring practices in APAC countries. The survey findings, based on opinions from key diabetes experts, revealed that glucose monitoring practices varied among the participating APAC countries, as well as between individuals with T1DM and those with T2DM. Among people with T1DM, 20%-100%, <10%-100%, and <10%-70% used quarterly HbA1c, daily SMBG, and weekly CGM, respectively. Japan reported the highest CGM usage. In contrast, Hong Kong reported <10% adoption for SMBG and CGM. Six other countries (China, India, Malaysia, the Philippines, Singapore, and South Korea) reported ≤10% continual use of CGM. India reported suboptimal utilization rates across all types of glucose monitoring, while Singapore reported that a higher proportion of individuals with T1DM (~30%) used CGM intermittently, with a frequent practice of using CGM prior to their doctor’s consultation, to aid in medical review.

Utilization rates for the three types of glucose monitoring were generally lower in T2DM than in T1DM. About 30%-100%, <10%-70%, and <10%-40% of individuals with T2DM used HbA1c, SMBG, and CGM, respectively, in the various APAC countries that participated in the survey. In Australia, Japan, South Korea, and Singapore, HbA1c utilization rates were generally ≥70%, while some settings in Australia, the Philippines, China, Japan, South Korea, and Malaysia reported ≥50% SMBG usage. In contrast, CGM adoption was more than 10% in only two countries: China and South Korea. One possible contributor to the lower glucose monitoring rates in T2DM versus T1DM individuals may be the type of HCP. For instance, in Australia, most people with T1DM see specialists or endocrinologists who may order CGM or more frequent testing than the primary care physicians seeing most individuals with T2DM. In addition, the Australian government has recently subsidized CGM for all individuals with T1DM, which may translate into higher uptake of CGM in this population versus those with T2DM. Furthermore, most individuals with T2DM receive regimens that do not need frequent glucose monitoring; this in turn may contribute to the low uptake of CGM.

### Frequency of Glucose Monitoring in APAC

For each type of glucose monitoring, testing frequency among people with T1DM or T2DM varied according to the treatment regimen: (1) multiple daily insulin injections (MDIs) or (2) basal insulin and/or oral glucose-lowering drugs (GLDs). In APAC, people with T1DM or T2DM on MDIs often have higher monitoring frequencies than those with T2DM on basal insulin and/or oral GLDs. Concurring with the common practice in APAC, current guidelines recommend HbA1c monitoring frequency in the range of two to four times annually (ie, every 3-6 months), adjusted to the level of glycemic control.^5,27,28^ In contrast, there are no strict recommendations on SMBG timing (eg, fasting, before and after meals and snacks, at bedtime, on hypoglycemia suspicion), although the frequency and testing pattern ultimately depend on the insulin regimen.^15,28^ Many individuals on MDIs need to perform SMBG six to ten times per day to help prevent hypoglycemia or hyperglycemia and adjust insulin doses and diet/lifestyle choices, but in some, this rate may be impractical, too costly, or distressing.^15,28^ For people with T2DM who do not require MDIs, evidence is limited regarding the optimal SMBG prescription,^15,27,29^ so a one-week structured SMBG may be more useful for HCPs than frequent or regular testing. In either case, CGM may aid the clinician by providing a more granular assessment (if on MDIs) or help guide lifestyle adjustments (for both insulin and non-insulin users).^15,28,29^

In current guidelines, the recommended frequency is continual use for rtCGM and scans at least every eight hours for isCGM.^15^ To determine the TIR, CGM should ideally be worn for at least 14 days and remain active ≥70% of the time.^6,22,30^ Given the varied CGM practices in APAC, we propose a daily isCGM scanning frequency—at least eight times in individuals with T1DM, and every four to six hours in those with T2DM on MDIs.

### Factors Influencing Glucose Monitoring Choices

Consistent with current guidelines, the type of diabetes and the insulin regimen are the principal factors influencing the choice and the frequency or timing of glucose monitoring among people with diabetes in APAC.^15,27-29^ Other factors include risk of hypoglycemia and baseline HbA1c: For individuals at high risk of hypoglycemia, SMBG and CGM may be needed in addition to the conventional HbA1c because HbA1c alone does not provide information on the occurrence of hypoglycemia episodes. For people with diabetes who have higher baseline HbA1c, more frequent pre-prandial monitoring may help assess premeal glycemia; those with lower levels may need less frequent monitoring, albeit directed postprandially.^31^

Non-clinical factors, such as government subsidy or reimbursement, also play a significant role in APAC glucose monitoring patterns. In Singapore and the Philippines, CGM costs are not reimbursed for both T1DM and T2DM, while in Australia, CGM costs became subsidized only in July 2022 for people with T1DM. Although South Korea approved CGM reimbursement in January 2019, it was only in August 2022 that it approved reimbursement for sensors and patient training. Therefore, prior to that, fewer people with diabetes used CGM versus SMBG. In Australia, the government also subsidizes people with T2DM, receiving insulin treatment, for the ongoing use of SMBG strips—but not for CGM usage. In Hong Kong, only HbA1c costs are reimbursed, resulting in low utilization rates for SMBG and CGM. In addition, the lack of CGM training among primary HCPs in China, Singapore, and the Philippines may have led to lower adoption of CGM versus HbA1C or SMBG in these countries.

## Patient Profiles Suitable for the Initiation of CGM

Multiple clinical trials have established the benefits of CGM among people with T1DM, including children, adolescents, adults, and pregnant women.^32-38^ Several studies have also demonstrated that regardless of the diabetes type (T1DM, T2DM, or gestational diabetes mellitus [GDM]), individuals who receive intensive insulin therapy (ie, MDIs) benefit from CGM.^33,39,40^ Furthermore, evidence suggests that CGM is significantly more effective than conventional blood glucose monitoring in lowering HbA1c in people with poorly controlled T2DM on less-intensive regimens (ie, basal insulin and/or oral GLDs).^41,42^ Based on current guidelines, people with diabetes with severe hypoglycemia, hypoglycemia unawareness, or any problematic hypoglycemia (ie, unexplainable, recurrent, asymptomatic, or nocturnal) constitute another population who would benefit from CGM, especially if the system includes a real-time low-glucose alarm.^15,22,30,43,44^

While all these people with diabetes will predictably benefit from CGM, resource limitations in APAC may necessitate identification and prioritization of specific groups who will benefit the most from CGM. The foremost APAC population that is suitable for CGM initiation are people with diabetes who need long-term monitoring, such as individuals with T1DM or T2DM who are on MDIs with suboptimal glycemic control or those with recurrent or severe hypoglycemia, in accordance with current guidelines.^22,30^ Other key patient profiles in APAC that may be suitable for CGM initiation include the following:

- People with diabetes who are on renal replacement therapy—Currently, the use of CGM is not approved in patients with dialysis. However, evidence suggests that CGM correlates with HbA1c and SMBG among people with diabetes and severe CKD, including those on hemodialysis.^45,46^ Although the amplitude of glycemic variability may differ between on- and off-dialysis days, as well as between pre- and post-dialysis days,^46,47^ there is growing evidence that CGM detects this variation as well as asymptomatic hypoglycemia while also reducing time below range in these individuals.^45-47^ Furthermore, in individuals with end-stage renal disease, TIR provides crucial information that cannot be fully captured by either HbA1c or glucose management indicator.^48^
- People with diabetes who are fasting during Ramadan—Muslim communities in APAC constitute almost two-thirds of the world’s Muslim population.^49^ Intermittent fasting during Ramadan can increase the risk of acute diabetes complications in some people with diabetes. These complications include hypoglycemia, hyperglycemia, dehydration, and ketoacidosis.^50^ Owing to the potentially increased glycemic variability in this population cohort, CGM may be the preferred choice over SMBG for individuals with T1DM who are fasting during Ramadan.^51^ Continuous glucose monitoring can serve as a useful tool for monitoring glycemic variability during Ramadan fasting. The use of CGM has been associated with a reduction in HbA1c levels and a decrease in the incidence of complications associated with fasting.^52,53^
- People with diabetes who are hospitalized or isolated in the community for coronavirus disease 2019 (COVID-19) and requiring remote glucose monitoring—Emerging evidence from small-scale observational studies has illustrated the benefits of inpatient CGM for those with COVID-19, including early detection of hypoglycemia and hyperglycemia, more timely decision-making in diabetes, reduced work burden and exposure risk for health care staff, and lower consumption of personal protective equipment.^54^ Moreover, CGM with remote-monitoring features has facilitated remote diabetes monitoring and individualized home care, thus controlling the risk of viral transmission despite pandemic-related disruptions in health care delivery.^55^
- Women with GDM and pregnant women with pre-existing diabetes—GDM is highly prevalent in APAC settings and is linked to increased health risks for both the mother and the baby.^56^ CGM can identify specific patterns of hyperglycemia throughout the day, which may assist in anticipating the occurrence of maternal-fetal complications and the likelihood of the need for pharmacological interventions.^57^ In pregnant women with T1DM receiving intensive insulin therapy, CGM use is associated with better neonatal outcomes, presumably due to reduced exposure to maternal hyperglycemia.^58^

Table 2 provides a comprehensive list of the potential patient groups in APAC that may benefit from CGM initiation.

**Table 2.** Profiles of People Living With Diabetes Suitable for Consideration of CGM Initiation, Based on a Survey of 15 Diabetes Experts From APAC.

Key patient profiles^a^
People with diabetes on MDIs or complex insulin regimens, especially those who are: • in need of long-term glucose monitoring • on renal replacement therapy^b^ • fasting during Ramadan^b^ • hospitalized or isolated in the community for COVID-19 and would benefit from remote glucose monitoring^b^ • undergoing cancer therapy • pregnant • with frequent or disabling hypoglycemia • at risk of or at high suspicion of nocturnal hypoglycemia
Other patient profiles^c^
• People with diabetes who are initiating insulin regimen (basal, premix or basal bolus) or undergoing intensification (eg, from basal to premix or MDI) • People with diabetes on high-risk oral GLDs (eg, sulphonylureas or glinides) • People with diabetes who are switching over from oral GLDs to any insulin regimen or undergoing change in oral GLD regimen • Patients who have undergone pancreatectomy or organ transplant • People with diabetes who are on corticosteroid therapy • People with diabetes who have comorbid cardiovascular conditions • Women with gestational diabetes mellitus • Elderly people with diabetes • Children and adolescents with diabetes who would benefit from closer monitoring • Any patient suspected of having a discrepancy between HbA1c levels and SMBG readings • People with newly diagnosed T2DM and not achieving target HbA1c • People with prediabetes or impaired fasting glucose who would benefit from constant feedback to motivate behavioral change • People with diabetes who have infections • People with diabetes who are non-adherent to optimal lifestyle and dietary measures • Hospitalized or surgical patients with diabetes

APAC, Asia-Pacific; CGM, continuous glucose monitoring; COVID-19, coronavirus disease 2019; GLD, glucose-lowering drug; HbA1c, glycated hemoglobin; MDI, multiple daily insulin injection; SMBG, self-monitoring of blood glucose; T2DM, type 2 diabetes mellitus.

a In order of decreasing numbers of survey responses.

b With equal numbers of survey responses.

c In no particular order.

## Patient Profiles Suitable for Long-Term Use of CGM

Continual or long-term use of CGM should be individualized based on the patient’s needs, preferences, and economic constraints. As in current guidelines, people with diabetes receiving MDIs or insulin pump therapy are the main population suitable for long-term CGM use.^15,22,30,43,44^ Another key group in APAC that could potentially benefit from long-term CGM use are people with diabetes for whom SMBG is not feasible (eg, those with needle phobia or occupational restrictions). In a recent cross-sectional study among people with T1DM aged 13 to 19 years, 84% found isCGM less painful than SMBG, while 90% of subjects reported no pain with isCGM application or sensor scanning.^59^ Similar findings have been reported with rtCGM,^60^ supporting the viability of CGM as an option for people with diabetes with fear of pain. Meanwhile, a single-arm prospective study has recently shown that HbA1c improved after CGM initiation among people with T1DM who have baseline HbA1c >9% and a history of SMBG non-adherence.^61^ In APAC, this special population represents another key patient profile suitable for long-term CGM use. Several randomized controlled trials, including DIAMOND, GOLD, and Flash UK, have established the benefits of CGM in individuals with T1DM and high HbA1c levels.^36,38,62^ Flash UK is an open-label, multicenter, parallel-group, randomized controlled trial that aimed to evaluate the impact of flash glucose monitoring versus SMBG in people with T1DM and suboptimal glycemic control. The study established that the use of flash glucose monitoring with optional alarms for high and low blood glucose levels is associated with significantly lower HbA1c levels when compared with the levels monitored by SMBG.^62^ Similarly, significantly greater reduction in HbA1c has been reported with rtCGM versus conventional blood glucose monitoring in individuals with T1DM and elevated HbA1c levels in the DIAMOND and GOLD studies.^36,38^

Another population cohort that may benefit from CGM use includes individuals with T1DM with severe hypoglycemia or unawareness of hypoglycemia. In the HypoDE randomized controlled trial, the use of rtCGM was associated with a significant reduction in the incidence of hypoglycemic events in people with T1DM on MDIs and severe hypoglycemia or impaired awareness of hypoglycemia.^37^ Another randomized, parallel-group study (I HART CGM) showed that transitioning from flash to rtCGM that features alarms and alerts has a significant positive effect on hypoglycemia outcomes, and that the ongoing use of rtCGM sustains this effect in high-risk patients, regardless of the hypoglycemia thresholds.^63^ A separate study on 15 000 rtCGM users with “Urgent Low Soon” (ULS) alert being used optionally found that the use of the predictive ULS alert was associated with notable reductions in both clinical and biochemical hypoglycemia as well as time spent in clinical hypoglycemia. This effect was independent of the frequency of screen views.^64^ Table 3 provides a comprehensive list of the potential patient groups in APAC suitable for CGM continuation.

**Table 3.** Profiles of People Living With Diabetes Who Are Suitable for Long-Term CGM Use, Based on a Survey of 15 Diabetes Experts From APAC.

Key patient profiles^a^
People with diabetes: • on MDI regimens • on insulin therapy, with high risk of hypoglycemia • on insulin therapy, with impaired awareness of hypoglycemia • with baseline HbA1c above 9% and non-adherent to SMBG
Other profiles^b^
People with diabetes: • in whom SMBG is not feasible (needle phobia/occupational reasons) • on basal insulin with or without oral GLDs • on oral GLDs

APAC, Asia-Pacific; CGM, continuous glucose monitoring; GLD, glucose-lowering drug; HbA1c, glycated hemoglobin; MDI, multiple daily insulin injection; SMBG, self-monitoring of blood glucose.

a In order of decreasing numbers of survey responses.

b In no particular order.

## Benefits of CGM

The clinical benefits of CGM in people living with diabetes are well established in interventional and real-world studies, although more studies have been conducted in people with T1DM than with T2DM.^15,22^ Large-scale and multicenter clinical trials have demonstrated that rtCGM and isCGM are both beneficial for improving HbA1c and reducing time in hypoglycemia.^36-38,65-68^ The multicenter, prospective COACH study showed that non-adjunctive rtCGM use in diabetes management may result in significantly fewer severe or debilitating hypoglycemic events in individuals with T1DM or T2DM requiring insulin.^68^ These CGM benefits have been confirmed by a recent narrative synthesis of 32 systematic reviews and meta-analyses throughout the years.^69^ Other outcomes that have improved with CGM use include time in hyperglycemia, hospitalization related to diabetes emergencies, quality of life, quality-adjusted life years, diabetes-specific quality of life (diabetes distress, hypoglycemic confidence), and treatment satisfaction rates.^69-72^ Among children and youth with diabetes, CGM use has also exhibited improved adherence, better parental sleep, less family and psychosocial stress, and higher acceptability over SMBG.^59,69^ Furthermore, remote data sharing of CGM data enables parents or caregivers to receive automated alerts for hypoglycemia.^20^

The benefits of CGM are largely similar across the globe. In the pre-meeting survey, the most common outcome perceived to improve with CGM versus SMBG was hypoglycemia in T1DM and TIR in T2DM. Hyperglycemia was also a key perceived clinical benefit of CGM over SMBG, regardless of the diabetes type.

## Challenges to Optimization of Use of CGM in APAC Settings

The key challenges to optimizing CGM initiation in APAC were reported as: (1) financial costs, (2) social stigma, (3) lack of evidence in specific areas, and (4) lack of awareness among HCPs and people with diabetes, especially those with T2DM. Meanwhile, the top barriers to CGM continuation include financial costs, poor utilization of CGM data, and concerns regarding accuracy.

First, as highlighted in the previous sections, financial cost continues to affect CGM uptake in APAC. For instance, the non-reimbursable–only market remains a significant barrier to access in South Asia and Southeast Asia, as does the limited market entry of more advanced CGM technology.^73^ In India, full utilization of diabetes technologies is curtailed by high initial consumer costs, which are required to upgrade local protocols, yet aggravated by lack of insurance coverage.^74^ In Australia, the recent subsidization of CGM for all individuals with T1DM has helped to address the cost barrier and enable much greater equity of health care access in this group. For individuals with T2DM, however, the prohibitive CGM costs remain an unmet challenge.^75^ Second, although clinicians assert that social stigma on diabetes is becoming less relevant in APAC, issues about the application of glucose sensors on prominent body parts still exist (eg, preference for leg or abdomen instead of arm). Finally, issues on accuracy and lack of adequate evidence still exist in APAC. Health authorities need to address these issues when implementing any health technology as prescribed in Western guidelines.^76^

## Potential Solutions for Optimization of Use of CGM

### HCP-Related Initiatives

The generation of more clinical data and, as feasible, local evidence is a top priority toward enhancing CGM utilization in APAC. These endeavors help convince clinicians of CGM benefits, which international clinical trials have already proven. Real-world evidence (RWE) may also encourage CGM subsidization or reimbursement by the government or private insurance companies. One such study demonstrating the real-world effectiveness of CGM was a retrospective cohort study in insulin-treated individuals with diabetes, where rtCGM showed significant improvement in HbA1c and reduction in emergency department visits and hospitalizations for hypoglycemia.^77^ Since the United States passed the 21st Century Cures Act in 2016, health agencies have advocated for RWE generation to support rapid development and approval of medical innovations.^78^ Accordingly, the ADA and the European Association for the Study of Diabetes (EASD) have called on international and national research organizations to collect RWE on diabetes technologies.^76^

Cost-effectiveness analyses (CEAs) may also facilitate CGM financing. Early evidence points to the cost-effectiveness of CGM when used long term and regularly in people with diabetes who have high baseline HbA1c or are experiencing frequent hypoglycemia.^79,80^ A CEA by Jendle et al concluded that flash glucose monitoring (with the FreeStyle Libre system) is a cost-effective alternative to SMBG in individuals with T2DM who are treated with insulin and unable to achieve their glycemic goals.^81^ Similarly, the DIAMOND trial also demonstrated the cost-effectiveness of the Dexcom CGM system in individuals with T1DM intensively treated with insulin.^82^ However, CEAs from developing countries are sparse, and the available data may not be generalizable across regional settings.^79^

Potential HCP-related solutions in APAC also include guideline development, medical education, and case compendiums, as well as allied-staff training. These initiatives concur with the recommendations of the ADA and EASD for professional societies, researchers, academicians, and other stakeholders in diabetes technology.^76^

### Patient-Related Initiatives

The ADA and EASD advise diabetes-technology consumers, including people with diabetes and their families, to actively seek and discuss information with their HCPs and provide feedback.^76^ Correspondingly, in APAC, the key patient-related initiatives to optimize CGM uptake involve the development of training videos on CGM use (including key pictorial summary outputs), patient education materials, patient testimonial videos or booklets, and patient forums or focus group meetings. Other patient-communication solutions include YouTube videos, websites with frequently asked questions, helplines, artificial intelligence–based tools, and social media platforms.

## Can CGM be the New Standard of Care for Glucose Monitoring?

The ADA and other Western and international medical societies recommend CGM for people with T1DM and for those with T2DM who are on intensive insulin regimens such as MDIs or insulin pumps.^15,22,28,30,43^ Furthermore, the American Association of Clinical Endocrinology prefers CGM over SMBG as a monitoring method.^16^ In these guidelines, CGM may be considered for people with T2DM on less-intensive insulin therapy, including those on basal insulin with or without oral GLDs.^15,22,28,30,43^ Nevertheless, more evidence is needed in this population and in people with T2DM who are receiving oral GLDs alone. Altogether, while CGM is becoming the new standard of care globally, glucose monitoring should be individualized according to the local context, availability of technology, and socioeconomic milieu.

## Future Directives

The standard of care in glucose monitoring for people living with diabetes is now transitioning from SMBG to CGM. Although the measurement of HbA1c in diabetes management remains relevant, CGM summary statistics (eg, glucose management indicator, TIR, time below range, glycemic variability) yield more detailed and actionable information for the managing physician, so they are increasingly utilized. Due to interregional and intraregional differences in policies and availability of resources, as well as limited quality studies undertaken in the APAC region, international guidelines may not be completely applicable to APAC. To optimize the use of CGM as the new standard of care for glucose monitoring, regional consensus guidelines that consider the APAC-specific setting and constraints should be formulated. As APAC represents one of the largest growing regions in terms of diabetes prevalence, there is an urgent need and a call to action for greater collaboration for the generation of RWE and CEAs from the APAC region. Given the vast amount of CGM-generated data, the development of a tool that can utilize this data and guide informed clinical decisions may be the key to precision medicine in future.
